# CT imaging features and diagnostic algorithm for hepatic cystic echinococcosis

**DOI:** 10.1038/s41598-025-94860-y

**Published:** 2025-03-28

**Authors:** Hao Zhang, Li Zhang, Chi Zhang, Yan-hao Zhu, Yi-en Hong, Lin Li, Li Lai

**Affiliations:** 1 Department of Radiology, Dianjiang People’s Hospital of Chongqing, Chongqing, 408300 People’s Republic of China; 2 Department of Radiology, Changdu People’s Hospital of Xizang, Xizang, 854000 People’s Republic of China; 3 Class 21, Grade 2025, Chongqing Yangjiaping Middle School, Chongqing, 400050 People’s Republic of China; 4 Department of Pharmacy, Dianjiang People’s Hospital of Chongqing, 116 North Street, Guixi Street, Dianjiang County, Chongqing Municipality, 408300 People’s Republic of China; 5 Department of Radiology, Sichuan Academy of Medical Sciences & Sichuan Provincial People’s Hospital, 32# W. Sec 2, 1st Ring Rd, Qingyang District, Chengdu, 610072 Sichuan Province People’s Republic of China

**Keywords:** Cystic echinococcosis, Computed tomography, Pathology, Diagnostic algorithm, Liver diseases, Parasitic, Parasitic liver diseases, Computed tomography, Radiography

## Abstract

To systematically analyze CT imaging features of hepatic cystic echinococcosis (CE), explore radiological-pathological correlations, and develop a diagnostic algorithm for accurate disease classification. This retrospective study included 48 pathologically confirmed cases of hepatic CE from two medical centers. CT imaging features were analyzed by two experienced radiologists, evaluating lesion characteristics including location, morphology, wall features, and calcification patterns. Imaging findings were correlated with pathological results. A diagnostic algorithm was developed and validated, with inter-observer agreement assessed using Fleiss kappa coefficient. Seven distinct CT imaging patterns were identified, corresponding to different pathological stages: unilocular cystic (25.0%), multivesicular (8.3%), collapsed inner wall (10.4%), partially solidified (10.4%), solidified (16.7%), and calcified (25.0%) types, with complicated cases (4.2%) showing additional features. The proposed diagnostic algorithm achieved 94.0% accuracy (451/480 classifications) in validation testing by ten junior radiologists, with excellent inter-observer agreement (quadratic-weighted Fleiss kappa coefficient = 0.740 [95% CI 0.577–0.902], Gwet’s AC2 coefficient = 0.768). Primary diagnostic challenges involved differentiating between CE2 and CE3b lesions, and between CE3b and CE4 lesions. This study explores the correlation between CT imaging patterns and pathological stages of hepatic CE, proposing a validated diagnostic algorithm. The findings provide valuable insights for CE classification, particularly in regions where the disease is emerging or underrecognized.

## Introduction

Cystic echinococcosis (CE) is a severe zoonotic parasitic disease caused by the larval stage of Echinococcus granulosus, a tapeworm that primarily affects the liver. CE poses a significant global health challenge, affecting over one million people worldwide with an estimated annual healthcare cost of $3 billion^1^. The World Health Organization (WHO) has designated echinococcosis as one of the 17 neglected tropical diseases targeted for control or elimination by 2050^2^.

As a public health concern, CE is particularly prevalent in regions with intensive livestock farming, such as Central Asia, South America, and the Middle East. The annual incidence ranges from 1 to 200 cases per 100,000 individuals. The liver is the most commonly affected organ, accounting for over 70% of CE cases, with a mortality rate of 2–4%^1,2^. Furthermore, recent epidemiological studies have demonstrated an expanding geographic distribution of CE, attributed to factors such as globalization, increased population mobility, and changing patterns of pet ownership^3^. Advanced imaging equipment has revealed potentially underreported cases, yet diverse imaging manifestations of hepatic CE continue to pose diagnostic challenges in regions with limited disease awareness^4,5^.

Imaging modalities, including ultrasound, computed tomography (CT), and magnetic resonance imaging (MRI), play a crucial role in diagnosing and managing hepatic CE. These techniques provide valuable information on cyst location, morphology, and characteristics, as well as their relationship with surrounding tissues. Each of these modalities has its own strengths and limitations in lesion characterization^4^. CT offers certain advantages in terms of spatial resolution, density resolution, and multiplanar reconstruction capabilities^4,5^.

This study analyzed CT imaging findings from 48 pathologically confirmed cases of hepatic CE from two medical centers. The primary objectives were to (1) systematically categorize CT imaging manifestations of hepatic CE, (2) explore correlations between imaging features and pathological findings, and (3) develop and validate a CT-based diagnostic algorithm. The ultimate goal is to enhance diagnostic accuracy and streamline the evaluation process in regions where the disease is emerging or underrecognized^5^.

## Materials and methods

The study protocol was approved by the Institutional Review Board of Dianjiang People’s Hospital of Chongqing (Approval Number: DYLL-KY-2023-116). All methods were performed in accordance with the relevant guidelines and regulations, including the Declaration of Helsinki. Due to the retrospective nature of the study, the requirement for individual patient informed consent was waived by the Ethics Committee of Dianjiang People’s Hospital of Chongqing. All patient data were anonymized and de-identified prior to analysis to ensure privacy and confidentiality.

The study analyzed data from 48 cases of hepatic CE at two medical centers (Center A: 42 cases; Center B: 6 cases) located in or adjacent to endemic areas. All cases were confirmed by surgical pathology and serology between January 2023 and June 2024. Patients were included if they met the following criteria: (1) pathologically or serologically confirmed hepatic CE, (2) complete preoperative CT examination records, and (3) comprehensive clinical and follow-up data. Cases were excluded if there were concurrent hepatic diseases that could affect imaging interpretation, inadequate image quality, or incomplete clinical or pathological data. The final cohort consisted of 30 males and 18 females, aged 18 to 68 years (mean age: 40 ± 3.5 years). Forty patients presented with upper abdominal discomfort or palpable masses, while eight were asymptomatic and diagnosed incidentally. Forty patients had a documented history of living in pastoral areas.

All patients underwent CT examinations using either a GE 16-slice spiral CT (GE Healthcare, Chicago, IL, USA) or a Siemens 64-slice spiral CT scanner (Siemens Healthineers, Erlangen, Germany). Scanning parameters were standardized with a tube voltage of 120 kV, tube current of 200–300 mA, matrix of 512 × 512, and slice thickness and interval of 5 mm. Routine non-contrast scans were performed for all cases, with four cases receiving additional contrast-enhanced scans. For contrast-enhanced studies, a non-ionic iodinated contrast agent (Iohexol, 300 mg I/mL) was administered intravenously at 3 mL/s, with a total volume of 1.5 mL/kg body weight. Images were acquired during the arterial (25–30 s post-injection) and portal venous (65–70 s post-injection) phases.

Surgical specimens were processed following standard histopathological procedures. Tissues were fixed in 10% neutral buffered formalin for 24–48 h, embedded in paraffin, sectioned at 4–5 μm thickness, and stained with Hematoxylin and Eosin (H&E). Two experienced pathologists examined the stained sections under a light microscope, assessing the cyst wall structure, the presence of protoscoleces and daughter cysts, the inflammatory response in surrounding liver tissue, and the degree of fibrosis and calcification. Special stains, such as Periodic Acid-Schiff (PAS), were applied when necessary for confirmation.

Two radiologists with 20 and 21 years of experience in abdominal imaging independently reviewed all CT images, evaluating the lesion location and number, cyst size and shape, wall thickness and morphology, presence of calcifications, density of cyst contents, presence of daughter cysts or internal membranes, and signs of complications. Any discrepancies in interpretation were resolved by consensus. The detailed definitions and measurement criteria for CT imaging features are summarized in Table 1.

**Table 1:** Definitions and evaluation criteria for CT imaging features of hepatic CE.

Feature category	Parameter	Definition/measurement criteria
Wall characteristics	Thickness	Measured perpendicular to the wall at its thickest portion
	Regularity	Smooth: continuous, even contour
		Irregular: nodular or uneven contour
	Enhancement*	Present: visible wall enhancement on contrast-enhanced images
		Absent: no visible enhancement
Calcification	Pattern	Absent: no visible high-density areas
		Partial: scattered or incomplete high-density areas
		Complete: circumferential high-density rim or complete matrix calcification
Internal features	Cyst density	Measured in Hounsfield Units (HU) by placing ROI in largest homogeneous area
	Daughter cysts	Present: round or oval structures within main cyst
		Absent: no internal cysts
	Internal membranes	Present: linear structures floating within cyst
		Absent: no visible membranes
	Matrix	Homogeneous: uniform density throughout
		Heterogeneous: mixed density pattern
Morphology	Size	Maximum diameter measured in axial plane (cm)
	Shape	Round/oval: length-to-width ratio ≤ 1.5
		Irregular: ratio > 1.5
	Location	Liver segment and relationship to major vessels

*For cases with contrast-enhanced imaging.

Based on these imaging-pathological correlations, a proposed diagnostic algorithm was developed to systematically evaluate hepatic CE using CT characteristics. To validate this algorithm, ten junior radiologists with 1–3 years of experience in abdominal imaging, blinded to the final diagnosis, independently classified all cases. Their classifications were compared with the reference standard established by pathological findings.

Descriptive statistics were used to summarize the imaging and pathological findings. Continuous variables were expressed as mean ± standard deviation, and categorical variables as frequencies and percentages. Inter-rater reliability was assessed using quadratic-weighted Fleiss kappa coefficient (with 95% CI) and Gwet’s AC2 coefficient. Quadratic weighting was selected because misclassifications between non-adjacent CE types are considered clinically more serious than misclassifications between adjacent types. This weighting scheme appropriately penalizes larger disagreements (e.g., between CE1 and CE4) more heavily than minor discrepancies (e.g., between CE1 and CE2). Gwet’s AC2 coefficient was chosen for its advantages in multi-rater reliability assessment, including reduced sensitivity to base rates and marginal probability distributions, and superior handling of ordinal data such as CE classification types. All statistical analyses were performed using SPSS version 25.0 (IBM Corp., Armonk, NY, USA). The diagnostic algorithm flowchart was created using draw.io (version 21.6.8, JGraph Ltd., London, UK).

## Results

### Research population characteristics and basic CT findings

Among the 48 cases of hepatic CE analyzed in this study, CT imaging revealed diverse manifestations with distinctive characteristics. The general features of the lesions are summarized in Table 2.

**Table 2:** General characteristics of hepatic cystic echinococcosis lesions.

Characteristic	Category/measurement	Number of cases (%) or mean ± SD
Lesion distribution	Throughout liver	10 (20.8%)
	Right lobe	30 (62.5%)
	Left lobe	8 (16.7%)
Lesion number	Single	30 (62.5%)
	Multiple	18 (37.5%)
Lesion shape	Round or oval	44 (91.7%)
	Irregular	4 (8.3%)
Lesion size	Range	3.0–17.0 cm
	Mean ± SD	6.0 ± 3.5 cm
Wall thickness	Range	1–10 mm
	Mean ± SD	4.0 ± 2.1 mm
Cyst fluid density	Range	3–28 HU
	Mean ± SD	12 ± 5 HU
Calcification	Present	14 (29.2%)
CT imaging patterns		
Type 1	Unilocular cystic type	12 (25.0%)
Type 2	Multivesicular type	4 (8.3%)
Type 3a	Collapsed inner wall type	5 (10.4%)
Type 3b	Partially Solidified type	5 (10.4%)
Type 4	Solidified type	8 (16.7%)
Type 5	Calcified type	12(25.0%)
Type 6	Complicated type	2 (4.2%)

Note: HU = Hounsfield Units.

### Pathological findings

Histopathological examination revealed that hepatic CE consisted of a cyst wall and contents. The cyst wall was divided into an outer and inner layer. The outer layer formed a fibrous capsule due to the infiltration of epithelial cells, foreign body giant cells, and eosinophils. The inner layer comprised the parasite itself, with an outer keratin layer and an inner germinal layer. Microscopically, the keratin layer appeared as red-stained parallel laminar structures, providing protection and permeability. The germinal layer consisted of highly proliferative cells that formed brood capsules containing scoleces, which detached to form daughter cysts. The cyst fluid was mostly colorless or light yellow, containing antigenic proteins.

### CT and pathological correlation analysis

Table 3 summarizes the correlation between CT imaging patterns, pathological findings, and WHO-IWGE classification, revealing distinct patterns across different disease stages.

**Table 3:**
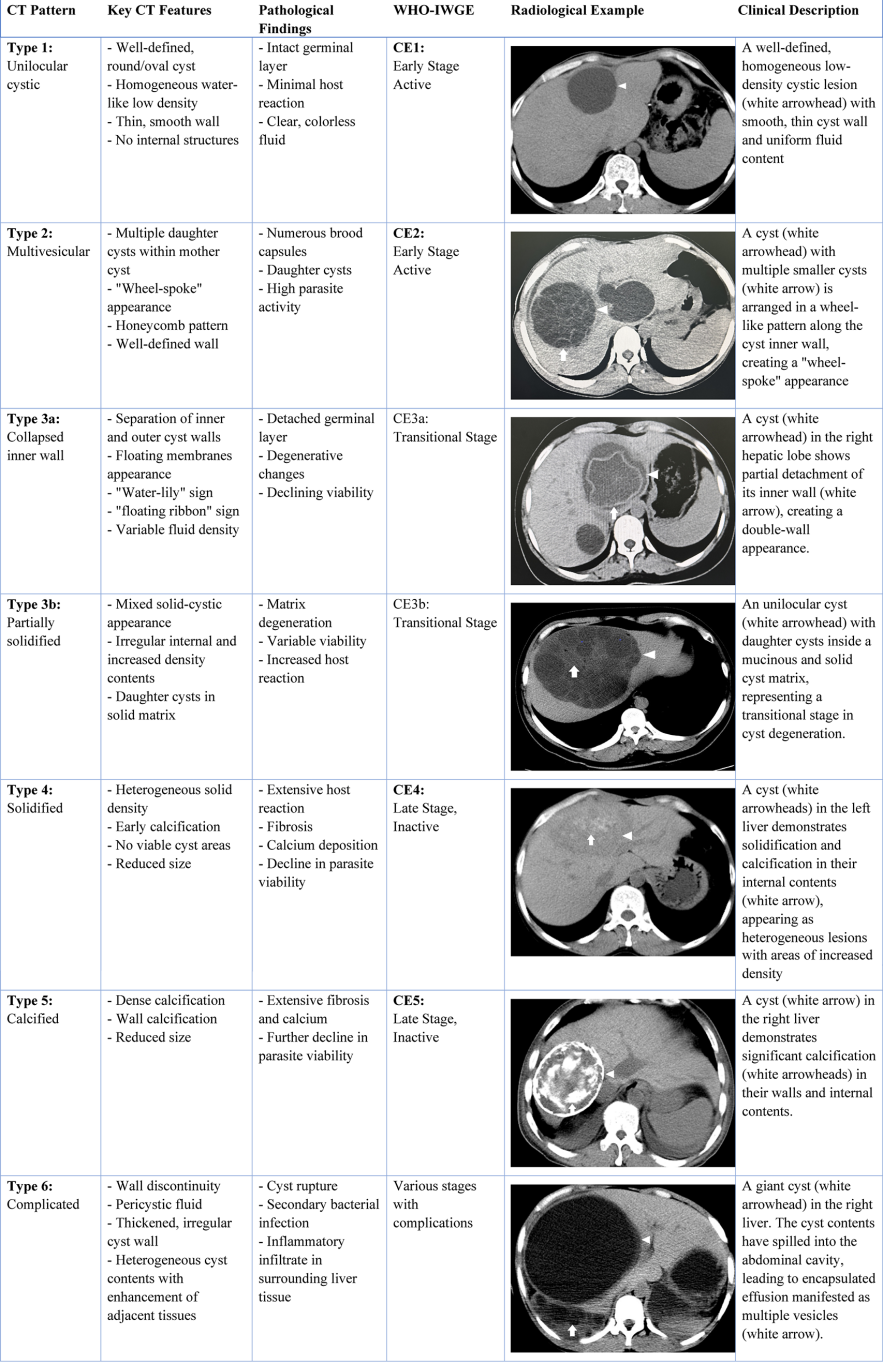
CT imaging patterns and corresponding pathological features with WHO-IWGE classification

Note: WHO-IWGE = World Health Organization Informal Working Group on Echinococcosis.

### CT-based diagnostic algorithm

**Fig. 1:**
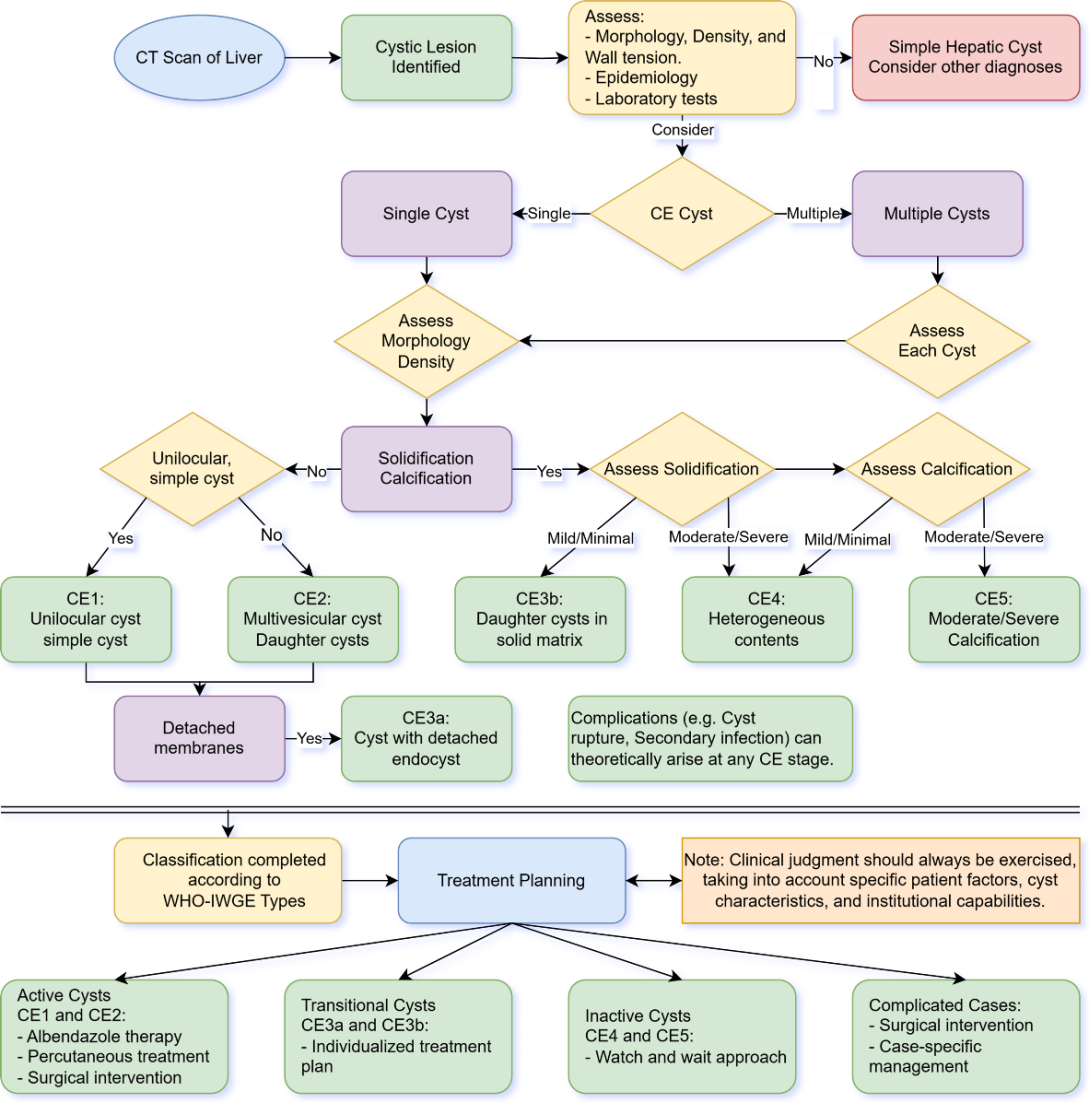
CT diagnostic algorithm for hepatic cystic echinococcosis based on CT findings.

The diagnostic algorithm based on CT findings incorporates key imaging features including cyst morphology, internal architecture, wall characteristics, and presence of calcification, while correlating these observed imaging patterns with disease activity. This structured approach allows for the classification of CE lesions according to their developmental stages and assists in clinical decision-making and treatment planning for patients with hepatic CE (Fig. 1).

### Algorithm validation results

The reliability of this classification system was validated through independent evaluation by ten junior radiologists. The detailed classification results and agreement analysis are presented in Table 4.

**Table 4:** Diagnostic performance in CT classification of hepatic cystic echinococcosis lesions.

CT pattern type	Reference standard*	Correct classifications†	Accuracy (%)	Most common misclassification
Type 1 (CE1)	12	115/120	95.8	Type 3a (3 cases)
Type 2 (CE2)	4	37/40	92.5	Type 3b (2 cases)
Type 3a (CE3a)	5	46/50	92.0	Type 1 (2 cases)
Type 3b (CE3b)	5	44/50	88.0	Type 2 (4 cases)
Type 4 (CE4)	8	75/80	93.8	Type 3b (3 cases)
Type 5 (CE5)	12	115/120	95.8	Type 4 (3 cases)
Type 6 (Complicated)	2	19/20	95.0	Type 3b (1 case)
Overall	48	451/480	94.0	–

Note: 1. Inter-rater reliability showed excellent agreement with quadratic-weighted Fleiss kappa coefficient = 0.740 (95% CI: 0.577–0.902) and Gwet’s AC2 coefficient = 0.768 (< 0.20: poor; 0.21–0.40: fair; 0.41–0.60: moderate; 0.61–0.80: good; > 0.80: very good).

2. * Reference Standard: Number of cases in each type based on pathological findings.

3. † Number of correct classifications by ten junior radiologists (total attempts = number of cases × 10 observers).

### Key findings

1. Primary classification challenges: • Between Type 2 and Type 3b lesions (4 cases misclassified as Type 2 when Type 3b; 2 cases misclassified as Type 3b when Type 2) • Between Type 3b and Type 4 lesions (3 cases misclassified as Type 3b when Type 4)
2. Accuracy rates: • Highest: Type 1 and Type 5 (95.8%) • Lowest: Type 3b (88.0%) • All types achieved accuracy > 88%

## Discussion

The present study provides an analysis and exploration of hepatic CE, integrating both imaging and pathological perspectives. From a clinical perspective, these disciplines are closely interconnected—pathology provides microscopic visualization while imaging offers macroscopic representation of pathological changes, with imaging manifestations closely reflecting the underlying pathological progression. This integrated approach enables relatively comprehensive characterization of disease manifestations and has potential value in parasitic diseases like CE where the pathological stage directly impacts clinical management^6–9^.

The pathological progression of CE is well-reflected in imaging findings. The anatomical distribution of portal blood flow explains the predominance of right lobe involvement (62.5% of cases)^4,10^. Hepatic CE typically manifests as large cysts with a mean diameter of 6 cm, a feature attributed to both its slow progression and delayed clinical presentation^7,8^. The cyst walls characteristically present as smooth and tense structures with an average thickness of 4 mm, with variations depending on multiple factors including disease duration, aging, infection, and trauma—these same factors can also lead to structural changes such as local collapse, shrinkage, deformation, and reduced wall tension^6,9^.

The unilocular cystic type corresponds to early-stage CE with intact germinal layers and minimal host reaction, typically appearing as well-defined, round lesions with homogeneous water-like density and thin, smooth walls. The average density measures 12 HU, which is intermediate between hepatic parenchyma and water (0 HU)^4,10^. CT-derived fluid density measurements correlate with pathological protein content and cellular debris found during examination, potentially serving as a biomarker for predicting procedure-related anaphylactic risks^8,9^. The multivesicular type demonstrates high parasite activity evidenced by numerous brood capsules pathologically, creating characteristic “wheel-spoke” and honeycomb patterns on imaging. The transitional stages, represented by collapsed inner wall and partially solidified types, show germinal layer detachment and increased matrix density, with separation of inner and outer cyst walls creating “water-lily” or “floating ribbon” signs. Late-stage disease is characterized by solidified and calcified types with extensive host reaction, fibrosis, and calcification patterns within cyst walls and internal structures. These calcification patterns, confirmed histologically, indicate disease chronicity and host immune response, thus providing a non-invasive method for assessing disease progression^4,6^. The degeneration of Echinococcus granulosus leads to necrotic changes and purulent fluid transformation. Although complications could theoretically occur at any CE stage, both documented complications (cyst rupture and infection) were exclusively observed in CE1-type lesions, suggesting an increased risk associated with this particular stage^8,9^.

The CT diagnostic algorithm (Fig. 1) provides a systematic approach for evaluating hepatic CE based on key imaging characteristics. This algorithm prioritizes readily identifiable CT features as initial decision points, including cyst wall characteristics, internal architecture, and calcification patterns^9^. These parameters were selected based on their strong correlation with pathological stages and consistent recognition across different observers. The presence of daughter cysts serves as a reliable indicator of active disease (CE2), while complete calcification suggests inactive status (CE5). The stepwise decision tree structure provides particular benefits for less experienced radiologists and clinicians by systematically guiding their classification decisions. Validation testing with ten junior radiologists showed a diagnostic accuracy of 94.0% (451/480 classifications) when compared with the pathological reference standard. The inter-observer agreement, assessed by both quadratic-weighted Fleiss kappa coefficient (0.740; 95% CI: 0.577–0.902) and Gwet’s AC2 coefficient (0.768) indicates the reproducibility of this algorithm. Misclassification patterns clustered around two main scenarios: differentiation between CE2 and CE3b lesions, and between CE3b and CE4 lesions. In CE2 versus CE3b misclassifications, the multicystic appearance often led to CE2 categorization, despite the presence of partial solidification that would indicate CE3b. For CE3b versus CE4 differentiation, subtle calcification within solidified components occasionally escaped detection. However, limitations emerge in transitional stages where imaging features show overlap, requiring detailed attention.

While the CT diagnostic algorithm demonstrates promising results in classifying hepatic CE lesions, to optimize patient care, it is important to recognize that different imaging modalities contribute unique advantages to the diagnostic process. Ultrasound is recommended as the first-line examination in endemic regions due to its advantages of being convenient, affordable and radiation-free^11^. The WHO-IWGE classification system based on ultrasound features provides standardized criteria for diagnosis and treatment planning^12^. In addition, contrast-enhanced ultrasound (CEUS) has shown great value in differentiating hepatic echinococcosis from other focal liver lesions, with diagnostic accuracy reaching 91%^13–15^. The characteristic “black hole sign” and rim-like enhancement pattern on CEUS helps distinguish hepatic alveolar echinococcosis from conditions like hepatocellular carcinoma and intrahepatic cholangiocarcinoma^16,17^. Intraoperative ultrasound (IOUS) provides real-time guidance during surgery. Compared to preoperative imaging, IOUS better demonstrates the relationship between cyst capsule and vessels, which is crucial for complete resection while avoiding complications^18^. IOUS is also more accurate in differentiating CE3b from CE4 lesions.

CT examination excels in detecting calcification and evaluating the relationship between lesions and surrounding structures^4^. The high spatial resolution of CT enables accurate assessment of vascular and biliary invasion^10^. MRI provides excellent soft tissue contrast and can better characterize cyst contents^19^. The “small vesicles sign” on T2-weighted imaging is highly specific for hepatic alveolar echinococcosis^10^. Diffusion-weighted imaging helps differentiate echinococcal lesions from malignancies, as the former typically show no diffusion restriction^20^. The enhancement of the capsule increases with delayed phases on contrast-enhanced scanning, and MRI is superior to CT in demonstrating this sign, which helps improve the detection rate of CE^4^. FDG-PET reflects lesion metabolic activity through FDG uptake induced by inflammatory responses^2122^. Recent studies have shown that CT, MRI and CEUS findings of microcystic structures, microcalcifications and blood supply correlate well with high metabolic activity on FDG-PET, suggesting their potential value in assessing disease activity^22–24^.

From a clinical perspective, the detailed characterization of CE lesions provided by this study can aid in diagnosis, staging, and treatment planning. The ability to differentiate between active, transitional, and inactive cysts based on CT features allows for more tailored management strategies^3,7,8^. For instance, active cysts (unilocular and multivesicular types) may benefit from albendazole therapy or percutaneous treatment, while inactive cysts (solidified and calcified types) might be suitable for a “watch and wait” approach, as suggested by current WHO guidelines^25^. Serial imaging helps monitor the dynamic nature of CE and assess disease progression.

Several limitations of this study warrant consideration. First, the retrospective design and relatively modest sample size (48 cases) may limit the generalizability of the findings. Second, while CT provides excellent morphological information, functional aspects of the disease that could be assessed through other imaging modalities were not evaluated. Third, the study lacks long-term follow-up data to assess how different imaging patterns might predict disease progression or treatment outcomes.

## Conclusion

This study explores the correlation between CT imaging features and pathological stages of hepatic CE. The proposed diagnostic algorithm, validated with an accuracy of 94.0% (451/480 classifications) and excellent inter-observer agreement (quadratic-weighted Fleiss kappa coefficient = 0.740 [95% CI 0.577–0.902], Gwet’s AC2 coefficient = 0.768), proposes a systematic approach for CE classification based on CT characteristics. These findings provide healthcare workers with valuable insights for assessing CE stages, which may improve clinical decision-making, particularly in regions where the disease is emerging or underrecognized.

## Data Availability

The data used to support the findings of this study are included within the article.
